# Etiology and Risk Factors for Splanchnic Vein Thrombosis in Non-Cirrhotic, Non-Neoplastic Patients: A Narrative Review

**DOI:** 10.3390/medicina61050933

**Published:** 2025-05-21

**Authors:** Mihaela Hostiuc, Ionut Negoi

**Affiliations:** 1 Internal Medicine, Department 5, Faculty of Medicine, Carol Davila University of Medicine and Pharmacy, 020021 Bucharest, Romania; 2 General Surgery, Department 10, Faculty of Medicine, Carol Davila University of Medicine and Pharmacy, 020021 Bucharest, Romania; ionut.negoi@umfcd.ro

**Keywords:** splanchnic vein thrombosis, portal vein thrombosis, non-cirrhotic, non-neoplastic

## Abstract

Splanchnic vein thrombosis (SVT) is a heterogeneous group of disorders affecting the portal, mesenteric, splenic, and hepatic veins. While frequently associated with liver cirrhosis and malignancy, SVT also occurs in non-cirrhotic, non-neoplastic patients. This narrative review evaluates the epidemiology and risk factors for SVT in this population. The prevalence and incidence of SVT in non-cirrhotic, non-neoplastic patients remain incompletely characterized, with estimates varying widely across studies. The clinical significance of SVT relates to potential complications, including intestinal ischemia, portal hypertension, and a possible underlying systemic disorder. Risk factors for SVT can be categorized into local abdominal conditions, thrombophilias, and systemic disorders. Local factors include inflammatory bowel disease, pancreatitis, abdominal surgery, and trauma. Thrombophilias, both inherited and acquired, are significant contributors to SVT risk. Systemic conditions associated with SVT include autoimmune disorders, pregnancy, hematological diseases, and infections. The complex interplay of these risk factors highlights the need for a comprehensive evaluation of SVT patients. Early recognition and management of these conditions can prevent potentially life-threatening complications and guide decisions regarding anticoagulation and long-term follow-up.

## 1. Introduction

Splanchnic vein thrombosis represents a spectrum of disorders affecting the veins from the splanchnic vein system (SVS), which includes portal vein thrombosis (PVT), superior and inferior mesenteric vein thrombosis (MVT), splenic vein thrombosis (SPVT), and Budd–Chiari syndrome (BCS), the latter being caused by hepatic vein thrombosis [1,2]. These thrombotic disorders may appear isolated or associated, involving multiple splanchnic venous segments. The most frequently cited location is the main portal trunk, followed by the superior mesenteric vein. Nevertheless, individual studies show a significant variability depending on specific conditions [3,4,5,6,7,8]. The extent of thrombosis is also highly variable, from non-occlusive to complete, with significant consequences for the clinical outcomes and management approaches [9].

The epidemiology of SVT in the general population remains incompletely characterized, with estimates of prevalence ranging widely across studies [10,11,12,13,14]. In patients with liver cirrhosis, the prevalence of PVT is around 13.92% [15], which suggests the presence of a significant prothrombotic status caused by or associated with advanced liver disorders. SVT also occurs frequently in patients with hematological malignancies, especially of the myeloproliferative type, such as polycythemia vera or essential thrombocythemia [16,17,18,19], being encountered in up to 65% of all patients with this complication [16]. However, SVT may also occur in patients without cirrhosis or malignancy, cases in which the etiology is less well-defined. The apparent scarcity of SVTs in non-cirrhotic, non-neoplastic patients may partly be caused by underdiagnosis, and the lack of severe clinical symptoms, especially in non-occlusive thrombosis. Many cases are discovered incidentally while performing imaging techniques for other indications [20,21,22].

The pathophysiology of SVT is similar to that of other vascular thrombosis, based on Virchow’s classical triad: venous stasis, endothelial injury, and hypercoagulability [23,24]. The splanchnic venous system is more prone to thrombosis due to slower blood flow, exposure to gut antigens, and proximity to abdominal inflammation. Thrombosis can be worsened due to local and systemic factors.

The clinical significance of SVT in non-cirrhotic, non-neoplastic (NC/NN SVT) patients is determined by their potential complications and effects on long-term outcomes. Acute complications include intestinal ischemia, acute bleeding, or infarction that could be life-threatening if not promptly recognized and treated. Chronic complications involve portal hypertension, variceal bleeding, ascites, and hypersplenism, which can significantly impair quality of life and require long-term management. SVT may be the first indication of an underlying and undiagnosed systemic condition, including oncological diseases, nephrotic syndrome, a thrombophilic disorder, or inflammatory disease.

This narrative review aims to evaluate the risk factors and epidemiology of SVT in non-cirrhotic, non-neoplastic patients.

## 2. Materials and Methods

We performed an electronic databases search, which included PubMed/MEDLINE, Scopus, and Web of Science, from their inception to March 2025. We used the following keywords: “splanchnic vein thrombosis”, “portal vein thrombosis”, “mesenteric vein thrombosis”, “splenic vein thrombosis”, “hepatic vein thrombosis”, and “Budd–Chiari syndrome”. These were combined with terms specifying the population of interest, such as “non-cirrhotic”, “non-neoplastic”, “without cirrhosis”, and “without cancer”. Additional terms related to etiology or risk factors were also included to ensure comprehensive capture of relevant literature.

In addition to the electronic database search, we performed a manual analysis of the reference lists from included studies and relevant review articles to identify additional eligible publications that the electronic search might have missed. We also tracked the citations of relevant articles to identify more recent publications citing the original works.

Given the anticipated heterogeneity in study designs, populations, interventions, and outcomes, a narrative synthesis approach was adopted rather than a formal systematic review or meta-analysis. This approach allowed for a comprehensive integration of diverse evidence while acknowledging the limitations of direct quantitative comparison across heterogeneous studies.

## 3. Results

### 3.1. Characteristics of the Epidemiology and Natural History of Splanchnic Vein Thrombosis in Patients with NC/NN SVT Compared to Patients with Cirrhotic and/or Neoplastic SVT

The epidemiology of splanchnic vein thrombosis in non-cirrhotic, non-neoplastic patients remains incompletely characterized, due to limited population-based data on incidence and prevalence. Most available information comes from hospital-based cohorts or specialized referral centers, which may not accurately represent the general population.

The estimated prevalence of SVT varies significantly across different studies. A meta-analysis conducted by Pan et al. found that in patients with liver cirrhosis (LC), the prevalence of portal vein thrombosis is 13.92%, with a 95% confidence interval between 11.81 and 16.91%. The prevalence of SVT among non-cirrhotic, non-neoplastic patients is less clear, but it appears to be considerably lower. A study examining data from the ENEIDA registry, which included 59,000 patients with inflammatory bowel disease (IBD), identified only 79 cases of SVT, indicating a relatively low prevalence even within this high-risk population [5]. However, this study likely underestimates the true prevalence, as many cases may remain undiagnosed due to the lack of specific symptoms, particularly in the early stages or with partial thrombosis [6,9,25].

The incidence NC/NN SVT is similarly difficult to ascertain with precision. Rajani et al. found the incidence and prevalence rates for PVT to be 0.7 and 3.7 per 100.000 inhabitants, respectively, but they also included patients with LC and malignancies [26]. In NC/NN SVTs, limited data suggest that the annual incidence is low, but specific high-risk populations demonstrate significantly higher rates. These include patients with known thrombophilia disorders [25], inflammatory conditions affecting the abdomen [5], infections (including COVID-19) [27,28,29,30,31,32], recent abdominal surgery [2], and pregnancy [33,34,35]. 

The incidence of NC/NN SVT does not appear to increase with age, although the incidence of venous thromboembolism (VTE) increases with age [36]. The evidence suggests that the incidence of NC/NN SVT is higher in younger individuals, particularly in those with thrombophilia. A study comparing cirrhotic and non-cirrhotic patients with PVT found that non-cirrhotic patients had a mean age of 57 years, while cirrhotic patients were older with a mean age of 65 years [37].

A 12-year study of the University of Chile found that the median age at diagnosis for non-cirrhotic PVT was approximately 57 years. Patients under 50 years old were more likely to have an underlying thrombophilic disorder [38]. Another study performed by Ogren et al. on 23,796 autopsies found a relatively normal distribution of portal vein thrombosis, with a mean age of 70.34 [39]. However, we have to consider, when analyzing these results, that this was a postmortem study.

Sex-specific differences in incidence are most likely minor. There are some studies indicating a slight male predominance, while others report no significant gender differences or even a slight female predominance [6,9,10,13]. Most likely, these demographic patterns may vary according to the underlying cause, with certain risk factors demonstrating different distributions by age and sex.

The natural history of untreated SVT in non-cirrhotic, non-neoplastic patients follows several potential trajectories. In some cases, spontaneous resolution occurs, particularly with partial thrombosis and when the precipitating factor is transient [20,40,41]. More commonly, however, untreated SVT persists and may extend to involve additional venous segments [10,42,43]. In one study of patients with inflammatory bowel disease and SVT, thrombosis persistence without significant changes was observed in 15% of cases, while progression occurred in 7% despite anticoagulation therapy [5].

Chronic SVT may lead to the development of collateral circulation as the body attempts to bypass the obstructed vessels. This process can result in cavernous transformation of the portal vein, characterized by the formation of multiple small venous channels around the occluded vein, a complication usually (but not exclusively) reported in neoplastic thrombosis of the SVS [44,45,46,47]. In the ENEIDA registry study, cavernous transformation was observed in 16% of IBD patients at the time of SVT diagnosis [5]. This finding indicates that many cases are diagnosed relatively late, after collateralization has already taken place, emphasizing the often-asymptomatic nature of the condition in its early stages.

The long-term sequelae of SVT include portal hypertension and its complications, such as bleeding, esophageal and gastric varices [48,49,50], ascites [50,51], and hypersplenism [52,53,54]. The development and severity of these complications depend on several factors, including the extent of thrombosis, degree of recanalization, the appearance of development of collateral circulation, or the functional status of the liver [9,50,51,55]. In patients without cirrhosis, the liver’s function is usually preserved, which may help mitigate some of the complications of portal hypertension. Ageno et al., in a study regarding the long-term clinical outcomes of SVTs (regardless of their cause), reported an incidence rate of 3.8 per 100 patient-years for significant bleeding and 5.6 per 100 patient-years for thrombotic events. In contrast, subjects with only transient risk factors for thrombosis experienced significantly lower rates of 0.5 and 3.2 per 100 patient-years, respectively [56].

Morbidity and mortality associated with SVT in non-cirrhotic, non-neoplastic patients are primarily related to complications from portal hypertension and, in cases of mesenteric vein thrombosis, intestinal ischemia [57]. In the ENEIDA cohort, no deaths were recorded during the thrombotic episode [5], suggesting that SVT may have a more favorable prognosis in non-cirrhotic, non-neoplastic patients compared to those with underlying liver disease or cancer. Interestingly, a study on survival after SVT, conducted on the Danish population between 1994 and 2013, found higher 30-day mortality rates following SVTs linked to non-neoplastic conditions. For instance, the 30-day mortality rate ratio for liver cancer was 1.7, for myeloproliferative neoplasms, it was 20, while for congestive heart failure it was 47.7 and 60 for atrial fibrillation and flutter [58].

Table 1 summarizes the relevant differences between SVT in patients with liver diseases (cirrhosis or liver cancer) and SVT in patients without liver diseases.

#### 3.1.1. Local Abdominal Conditions

SVT risk may also be increased by various local factors, such as inflammatory bowel disease, abdominal surgery, local trauma, etc.

Inflammatory bowel disease (IBD) [5], including Crohn’s disease [59,60,61] and ulcerative colitis [62,63,64,65], has been recognized as a significant risk factor for SVT in non-cirrhotic patients. The connection between IBD and venous thromboembolism (including recurrent VTE) is well-established, with an estimated two to three times increased risk compared to the general population [66,67]. This heightened risk extends to unusual sites of thrombosis, such as the splanchnic veins. The pathophysiology of this association has been shown to involve systemic inflammation [67,68], endothelial dysfunction [68,69,70], acquired prothrombotic changes in the coagulation system [68,71,72], platelet abnormalities [68,73], or impaired fibrinolysis [74,75]. Local factors like transmural inflammation [76,77,78], abscesses [60,66], and fistulas [60,79] may also directly impact neighboring vessels, promoting thrombotic processes.

The clinical characteristics of SVT in IBD patients offer insights into the nature of this association. In the ENEIDA cohort, SVT was predominantly associated with the active disease, suggesting that inflammation correlates with thrombotic risk [5]. The most frequently involved venous segments included the intrahepatic portal branches, the superior mesenteric vein, and the main trunk of the portal vein [5]. This distribution mirrors the anatomical relationship between the intestines and the splanchnic venous system, where intestinal wall inflammation could extend to nearby vessels.

Other inflammatory and infectious conditions of the abdomen represent essential risk factors for SVT. They include intra-abdominal abscesses (splenic, perirenal, or hepatic) [80,81,82,83,84,85,86], appendicitis [6,87,88,89], diverticulitis [90,91,92], cholecystitis [83,93,94], pancreatitis [81,95], or pelvic inflammatory disease [96,97,98,99,100].

Both acute and chronic pancreatitis deserves special consideration as causes of SVT. The intimate anatomical relationship between the pancreas and the splanchnic venous system, mainly the splenic vein, makes thrombosis a common complication of pancreatic inflammation or neoplasia [27,101,102]. Often, gallstones may be the underlying cause of acute pancreatitis leading to local venous thrombosis [103]. Alcoholic pancreatitis has been shown to cause local inflammation [104], leading directly to an increased risk of splenic or portal vein thrombosis [105,106]. In acute pancreatitis, inflammatory edema and necrosis can directly compromise the integrity of adjacent vessels [103,107], while the release of pancreatic enzymes may cause damage to the vascular endothelium [108]. In chronic pancreatitis, the incidence of splenic venous thrombosis is very high. For example, Agarwal et al. found a 22% incidence in 157 patients from a 20-year retrospective cohort [109]. The primary causes are local fibrosis and pseudocyst formation, which can lead to extrinsic compression of vessels, promoting stasis and thrombosis [103,109,110,111]. When the splenic vein is occluded, blood from the spleen may be redirected through the short gastric veins to the stomach, potentially leading to isolated left-sided gastric varices and gastric bleeding, even with preserved portal vein patency [112,113,114].

Abdominal surgical procedures and trauma represent another category of local risk factors for SVT. Specific surgical procedures are associated with a significantly higher risk, including splenectomy with devascularization, pancreatectomy with venous resection [115], and hepatic surgery (such as liver transplantation, hepatectomy in patients with LC, or other major hepatobiliary surgeries) [115,116,117,118]. The leading causes of this increased risk include direct surgical manipulation that may directly injure the veins, venous stasis associated with increased intra-abdominal pressure, postoperative inflammation, immobilization, and alterations in splanchnic blood flow [115,119,120]. Abdominal trauma, particularly blunt force injuries, may cause direct vascular damage or result in hematoma formation with extrinsic compression of vessels, potentially setting the stage for thrombosis [20,42,121,122].

Biliary disease, including cholecystitis [94,123,124], cholangitis [20,125], or biliary obstruction [42,43,126], has also been implicated in the development of SVT through both local and systemic mechanisms. Locally, the biliary tract’s inflammation can extend to nearby portal venous branches [104,127]. Biliary obstruction may cause portal vein edema and compression of intrahepatic venous structures, or it may lead to acute pancreatitis, which is associated with a high risk of SVT (particularly at the level of the splenic vein) [103]. The relationship between biliary disease and SVT appears to be bidirectional, with each condition potentially exacerbating the other through various pathophysiological pathways [128,129,130].

Intra-abdominal adhesions, resulting from previous surgery, inflammation, or infection, may contribute to SVT by causing vascular kinking, compression, or distortions [131,132]. These mechanical effects may decrease the blood flow, leading to stasis, which increases the risk of thrombosis. The risk of SVT related to adhesions may be significant in patients with multiple prior abdominal surgeries [20,42,133,134] or complicated intra-abdominal infections [135,136]. It should be noted that the relationship between intra-abdominal adhesions and venous thrombosis is also bidirectional, as venous obstructions have been shown to cause intra-abdominal adhesions [132,137].

Abdominal compartment syndrome is a disorder characterized by the presence of increased intra-abdominal pressure, which is prone to compromise organ perfusion. Abdominal compartment syndrome can increase the risk of SVT because it leads to a severe impairment of the venous return [138,139,140]. The heightened pressure directly compresses the splanchnic venous system, causing stasis and thrombosis [138]. The causes are varied, including major trauma, massive fluid resuscitation, abdominal surgery, and severe pancreatitis [141,142]. Early detection and decompression are essential for the prevention of SVT but also to diminish the risk of multiple organ dysfunction associated with this impaired perfusion.

Local conditions that increase the risk of NC/NN SVT are presented in Table 2.

#### 3.1.2. Thrombophilia

Thrombophilic conditions, characterized by an increased tendency to form blood clots, are significant risk factors for splanchnic vein thrombosis in non-cirrhotic, non-neoplastic patients [10,11,143,144,145]. These conditions can be classified as inherited (genetic) or acquired, with many patients presenting more than one thrombophilic abnormality. The role of thrombophilias in the pathogenesis of SVT is particularly important in the absence of other local abdominal factors. However, they may also co-work with local inflammatory or mechanical processes to promote thrombosis.

Inherited thrombophilias include a diverse group of genetic disorders altering various components of the coagulation and fibrinolytic systems. The Factor V Leiden mutation, which leads to resistance to activated protein C, is among the most common inherited thrombophilias in the Caucasian population [14]. This mutation results in excessive thrombin generation and a hypercoagulable state, increasing the risk of venous thromboembolism in general and superficial venous thrombosis in particular [1,6,7,14].

The prothrombin G20210A mutation, another prevalent genetic thrombophilia, results in elevated prothrombin levels and enhanced thrombin formation, similarly predisposing to thrombotic events [146,147,148,149]. Prothrombin G20210A has often been cited as an increased risk factor for SVT [1,6,7,14].

Deficiencies of natural anticoagulant proteins, including protein C, protein S, and antithrombin, represent another category of inherited thrombophilias relevant to SVT [1,7,8]. These deficiencies are relatively rare in the general population. However, they appear to be overrepresented in patients with SVT without cirrhosis or cancer, suggesting a potentially significant pathogenic role [150,151,152]. The prevalence of these deficiencies in SVT patients varies across studies [6,7,10,12,13,143]. However, a consistent finding is the frequent coexistence of multiple thrombophilic abnormalities in individual patients [12,143,153,154,155].

Mutations in the methylenetetrahydrofolate reductase (MTHFR) gene, particularly the C677T variant, have been associated with hyperhomocysteinemia and increased thrombotic risk, including in the splanchnic veins [13,14,153,156,157]. Increased homocysteine levels may favor thrombosis through increased endothelial damage [158,159], platelet aggregation [160], or abnormal coagulation factor activity [161,162,163]. Numerous studies have emphasized the relationship between MTHFR mutations, hyperhomocysteinemia, and SVT [13,18,156,157,164,165,166].

Acquired hyperhomocysteinemia, which is often caused by nutritional deficiencies (particularly of folate, vitamin B6, or vitamin B12), certain medications, or renal insufficiency [167,168,169], represents an acquired thrombophilic disorder relevant to SVT [170].

The clinical consequences of identifying thrombophilic conditions in patients with SVT are potentially significant. First, their presence may lead to clinical decisions regarding the duration and intensity of anticoagulation therapy, which should be more aggressive or increased in duration in subjects with clinically relevant thrombophilic abnormalities [56,171]. Second, it may guide family screening and genetic counseling for hereditary thrombophilia [172,173]. Third, it may optimize the management of specific conditions including pregnancy [174] or surgery [56,115,175], where the thrombotic risk might increase further. Fourth, it may prompt the investigation and management of associated conditions, such as vitamin supplementation for hyperhomocysteinemia or immunomodulatory therapy for antiphospholipid syndrome.

Nowadays, it is generally accepted that thrombophilia represents a significant contributor to SVT, so experts recommend systematic screening in affected subjects [153,171,176]. However, the optimal timing, extent, and specific components of such screening remain debatable. Ideally, thrombophilia testing should be conducted before initiating anticoagulation, as some tests (particularly those for lupus anticoagulant and protein C or S levels) may be influenced by anticoagulant therapy [177,178,179].

The most important thrombophilic conditions involved in the etiology of SVT are presented in Table 3.

#### 3.1.3. Hematological Malignancies and Clonal Diseases

Paroxysmal nocturnal hemoglobinuria (PNH), a rare bone marrow failure disorder characterized by complement-mediated hemolysis leading to anemia, peripheral blood cytopenias, and thrombosis [180], is another potential cause of SVT. The thrombotic tendency in PNH is particularly pronounced within the splanchnic circulation, with abdominal vein thrombosis being the initial feature in many cases [181]. In addition, thromboembolic disorders constitute a significant cause of mortality in PNH patients, accounting for up to two thirds of the direct related deaths [182]. The most important predictors of thrombosis-related mortality in this group were hepatic vein thrombosis (RR = 5.67), pulmonary embolism (RR = 5.7), mesenteric vein thrombosis (RR = 8.27), and venous stroke (RR = 4.87 [183]. The mechanisms behind this site specificity are not fully understood but may be linked to local complement activation and the unique vascular environment of the splanchnic circulation. The diagnosis of PNH should be considered for all patients with unexplained SVT, especially when accompanied by signs of hemolysis or unexplained cytopenia [180,181].

Myeloproliferative neoplasms (MPNs), including polycythemia vera, essential thrombocythemia, and primary myelofibrosis, represent a significant cause of SVT [155,184,185]. While these conditions technically fall under the category of neoplasms, which are outside the primary focus of this review, it is worth noting that MPNs can be occult at the time of SVT presentation, with normal or near-normal blood counts [16,17]. Therefore, even in patients without overt hematological abnormalities, evaluation for underlying MPN should be considered, particularly screening for the JAK2 V617F [155,184,186] or calreticulin [1,12,187,188] mutations. A notable proportion (up to 40%) of idiopathic SVT cases are associated with the JAK2 V617F mutation, pointing to an underlying MPN [189].

Platelet abnormalities, such as significantly increased platelet reactivity, have been linked to the pathogenesis of SVT [190]. The mechanisms behind this heightened platelet activity may involve enhanced adhesion to damaged endothelium [56,155,191], increased aggregation in response to agonists, including those related to undiagnosed myeloproliferative disorders [192], and increased release of procoagulant factor [153,191].

#### 3.1.4. Systemic Conditions

Beyond local abdominal factors, thrombophilic states, and hematological malignancies, various systemic conditions have been associated with splanchnic vein thrombosis in non-cirrhotic, non-neoplastic patients.

Systemic autoimmune and inflammatory diseases represent a significant category of conditions associated with increased risk of venous thromboembolism, including SVT. These disorders include systemic lupus erythematosus (SLE) [193], rheumatoid arthritis [194,195], vasculitis [83,196,197], and Behçet’s disease [81,198,199,200]. In addition to the aforementioned pathophysiological causes such as chronic inflammation, endothelial damage, or platelet activations, in these disorders, antiphospholipid antibodies, which may occur in isolation or association with other autoimmune diseases (particularly SLE), are known to contribute significantly to thrombotic risk [201,202]. These antibodies increase the thrombotic risk through various mechanisms, such as direct effects on the endothelial cells, inhibition of natural anticoagulant proteins, or activating platelets and complements [201,203,204]. Antiphospholipid syndrome (APS), characterized by persistent antiphospholipid antibodies and clinical manifestations of thrombosis, pregnancy loss, or other complications, has been identified as a risk factor for thrombosis at unusual sites, including SVT [6,81,205].

Behçet’s disease deserves special mention due to its strong association with venous thrombosis, including SVT. This multisystem inflammatory disorder, characterized by recurrent oral and genital ulcers, uveitis, and various cutaneous manifestations [206,207], carries a high risk of vascular involvement. The thrombosis in Behçet’s disease is thought to be primarily inflammatory rather than purely thrombotic [200,208,209], with histopathologic studies showing vasculitis of the vein wall [200,210]. Recognizing Behçet’s disease as a potential cause of SVT is particularly relevant in regions with a high patient population, such as the Middle East and East Asia [211,212].

Pregnancy and hormonal factors represent another category of systemic conditions associated with an increased risk for SVT. Pregnancy causes significant changes in the hemostatic system by altering the levels of particular coagulation factors (increases in factors VII, VIII, IX, X, and XII and von Willebrand factor (VWF); decreases in proteins S and C) and impairing fibrinolysis, collectively creating a hypercoagulable state [213,214,215,216]. This physiological adaptation, likely evolved to protect against postpartum hemorrhage [213], has side effects, including an increased risk of venous thromboembolism, even at unusual sites such as the splanchnic veins. A systematic review and meta-analysis of pregnant patients with non-cirrhotic portal hypertension reported significant maternal complications, including variceal bleeding (9.6%), ascites (2.3%), and severe anemia requiring blood transfusion (14.9%) [217].

Pregnancy outcomes in patients with pre-existing SVT or non-cirrhotic portal hypertension are also clinically significant. The same meta-analysis reported increased rates of adverse pregnancy outcomes, including spontaneous miscarriage (11.9%), gestational hypertension (4.5%), cesarean delivery (36.7%), and postpartum hemorrhage (4.7%) [217]. Fetal outcomes were similarly impacted, with higher rates of stillbirth (2.5%), preterm birth (21.6%), low birth weight (18.7%), and neonatal intensive care unit admission (15.5%) [217].

Hormonal contraceptives, particularly those containing estrogen, have been associated with increased risk of venous thromboembolism, including at unusual sites such as the splanchnic veins [218,219], with potentially lethal consequences [220]. The thrombotic risk appears to be related to estrogen dose and is more pronounced with earlier generations of oral contraceptives [221,222]. While the absolute risk of SVT associated with hormonal contraceptives is low, this risk may be substantially higher in women with other predisposing factors, such as inherited thrombophilias or inflammatory conditions [223]. In patients with thrombophilias such as factor V Leiden, the least thrombogenic oral contraceptives seem to be progestin-only variants, except for medroxyprogesterone acetate [224].

Hematological disorders, other than myeloproliferative neoplasms and clonal diseases, may also contribute to SVT risk. Hematological conditions also associated with SVT include sickle cell disease [45,225,226], thalassemia [227,228,229,230], and platelet disorders, either qualitative or quantitative [43,231].

Metabolic disorders and conditions may also influence SVT risk, although the associations are less well-established than other risk factors. Diabetes mellitus is known to be associated with a prothrombotic state [232,233]. The risk increase is moderate, but statistically significant, as shown by Li, who found an OR of 1.90 (CI 95% between 1.42 and 2.28) [233]. The presence of diabetes mellitus has been shown to increase the need of intestinal resection in patients with mesenteric vein thrombosis [234]. By the same meta-analysis, hypercholesterolemia has also been shown to increase the risk of portal vein thrombosis, with an OR of 3.59 (95% CI between 1.83 and 7.03) [233]. Similarly, obesity has been associated with multiple prothrombotic changes, including increased levels of fibrinogen and other coagulation factors, impaired fibrinolysis, and chronic low-grade inflammation. The association with an increased risk of SVT is less clear. The same meta-analysis has shown a non-statistically increased risk, with body mass index (BMI) being irrelevant [233].

Chronic kidney disease represents another systemic condition that may influence thrombotic risk, including in the splanchnic circulation. The relationship between kidney disease and thrombosis is complex, as renal dysfunction increases thrombosis through endothelial dysfunction [235,236], platelet alterations [237], inflammation [238], oxidative stress [238], and procoagulant and anticoagulant factors [239,240]. Additionally, the nephrotic syndrome, characterized by increased proteinuria, hypoalbuminemia, and edema, is associated with an exceptionally high thrombotic risk, thought to result from urinary loss of anticoagulant proteins, increased levels of procoagulant factors, and enhanced platelet reactivity [241,242,243,244]. Even if SVT is a rare complication of nephrotic syndrome [242,245], it is sometimes its first sign [246], and it is extremely important for the clinician to be aware of this potential association.

Non-abdominal infectious diseases may also contribute to the risk of SVT through systemic effects on the coagulation system [30]. This infection-induced hypercoagulability represents an evolutionary adaptation that helps contain pathogens while increasing thrombotic risk [247,248,249]. Specific infections associated with thrombosis include cytomegalovirus [11,29], HIV/AIDS [250,251,252,253], and various bacterial and parasitic infections. The increase in SVT associated with COVID-19 is also noteworthy. For instance, a study by Taquet et al. on a retrospective cohort of over half a million COVID-19 cases found the risk of VT after COVID-19 diagnosis to be 392.3 per million people, significantly higher than a matched cohort that received the mRNA vaccine (RR = 4.46) or patients with influenza (RR = 1.43) [254]. Additionally, in subjects with COVID-19, the mean age at which PVT occurred was considerably lower than the average age of PVT occurrence in patients with cirrhosis [255].

Systemic conditions that increase the risk of NC/NN SVT are summarized in Table 4.

## 4. Limitations

The limitations of our study reside in its narrative structure, as the heterogeneity of study design, study populations, interventions, and outcomes led to choosing this form of review instead of a systematic review or a meta-analysis. 

In addition, the epidemiology of SVT is hard to be defined because some cases remain undiagnosed due to non-specific symptoms or the etiology is not always searched properly. An international registry on SVT will probably solve this knowledge gap.

## 5. Conclusions

SVT is an unusual site of venous thromboembolism with non-specific symptoms making it difficult to diagnose outside the clinical spectrum of liver cirrhosis or hematological malignancies. Once the diagnosis of SVT is assessed, a review of common and uncommon causes must be done according to the patient’s characteristics. Finding and treating the cause together with the anticoagulant treatment will minimize the risk of complications and recurrence.

A better understanding of SVT epidemiology will enhance the ability of the clinicians to tackle this chameleonic vascular disease.

## Figures and Tables

**Table 1:** Differences between SVT in patients with liver diseases (cirrhosis or liver cancer) and patients with NC/NN SVT.

Feature	Aspect	With Liver Disease (Cirrhosis or Liver Cancer)	Without Liver Diseases
Prevalence and incidence	Prevalence	Higher prevalence especially in advanced LC	Lower prevalence. SVT is rare in individuals without liver diseases
	Incidence	Increases with progression of the disease	Lower, mostly related to prothrombotic systemic states (inherited or acquired such as MPNs)
Demographic	Age	Usually middle-aged to older adults	Wider age range: younger patients may be affected due to inherited thrombophilias
	Sex	Slight male predominance	No sex predominance
Risk factors	Local	Portal hypertension, decreased portal flow, structural changes in hepatic architecture, tumor invasion	Local inflammation (e.g., pancreatitis, abdominal infection), abdominal surgery, trauma
	Systemic	Coagulation abnormalities in cirrhosis with imbalance between pro- and anticoagulants Cancer-induced hypercoagulability	Important role of inherited and acquired thrombophilias (e.g., factor V Leiden, antiphospholipid syndrome, JAK2 mutation)
Diagnosis	Clinical presentation	Usually asymptomatic; symptoms related to liver disease	Often acute onset with abdominal pain or other gastrointestinal symptoms
	Imaging diagnosis	Often incidentally found during routine imaging tests (ultrasound, CT) on follow-up visits	Targeted imaging test due to acute presentation
Natural history	Progression	In cirrhosis may remain unchanged or resolve spontaneously. In liver cancer it usually progresses	More likely progresses if thrombosis and risk factors (e.g., local inflammation) are left untreated. Spontaneous resolution may occur in non-occlusive thrombosis caused by a transient risk factor.
	Recurrence	Possible, especially in decompensated cirrhosis and treatment failure of liver cancer	The risk varies according to risk factors (higher in inherited thrombophilia; lower in SVT provoked by local, transient factors (e.g., cholecystitis, pancreatitis)
Morbidity	Portal hypertension Ischemic complications	Worsens portal hypertension in patients with cirrhosis, leading to variceal bleeding, ascites, hepatic decompensation. Cavernous transformation of portal vein has been reported mainly in neoplastic SVT.	In the acute presentation may lead to acute mesenteric ischemia and infarction. If left untreated may lead to secondary portal hypertension.
Mortality		Rarely causes death unless complicated by massive variceal bleeding. Depending on the stage of the cancer	Higher in acute extensive mesenteric ischemia Lower if the underlying risk factor is treatable

LC—liver cirrhosis; SVT—splanchnic vein thrombosis; MPNs—myeloproliferative neoplasms; CT—computer tomography.

**Table 2:** Local conditions involved in the etiology of NC/NN SVT.

Local Abdominal Conditions	Examples
Inflammatory bowel diseases	Crohn’s disease, ulcerative colitis
Biliary diseases	Cholecystitis, cholangitis, biliary obstruction
Inflammatory and infections conditions	Pancreatitis, appendicitis, diverticulitis, pelvic inflammatory disease
Abdominal abscess	Hepatic, renal, splenic
Abdominal surgery	Splenectomy, hepatic surgery, pancreatectomy
Abdominal trauma	
Intraabdominal adhesions	
Abdominal compartment syndrome	Secondary to trauma, massive fluid resuscitation, abdominal surgery, severe pancreatitis etc

**Table 3:** Thrombophilias involved in the etiology of NC/NN SVT.

Thrombophilia	Examples
Inherited	Factor V Leiden mutation Prothrombin G20210A Deficiencies of natural anticoagulants (protein C, protein S, antithrombin) MTHFR gene mutations
Acquired	Antiphospholipid antibody syndrome Acquired hyperhomocysteinemia Acquired deficiencies of natural anticoagulants (protein C, protein S, antithrombin)

**Table 4:** Systemic conditions involved in the etiology of NC/NN SVT.

Systemic Conditions	Examples
Systemic autoimmune and inflammatory diseases	Systemic lupus erythematosus Vasculitis Behcet disease
Hormone-related conditions	Pregnancy Oral contraceptives Hormone replacement therapy
Hematological disorders	Myeloproliferative neoplasms (polycythemia vera, essential thrombocythemia, myelofibrosis) Paroxysmal nocturnal hemoglobinuria Sickle cell disease Thalassemia Platelet disorders
Metabolic diseases	Diabetes mellitus Hypercholesterolemia Obesity
Chronic kidney disease	
Non-abdominal infectious diseases	COVID-19 Various viral, bacterial and parasitic infectious

## Abbreviations

The following abbreviations are used in this manuscript:

SVT	splanchnic vein thrombosis
SVS	splanchnic vein system
PVT	portal vein thrombosis
MVT	mesenteric vein thrombosis
SPVT	splenic vein thrombosis
BCS	Budd–Chiari syndrome
NC/NN	non-cirrhotic, non-neoplastic
LC	liver cirrhosis
IBD	inflammatory bowel disease
COVID-19	coronavirus disease 2019
VTE	venous thromboembolism
MTHFR	methylenetetrahydrofolate reductase
APS	antiphospholipid syndrome
PNH	paroxysmal nocturnal hemoglobinuria
MPNs	myeloproliferative neoplasms
SLE	systemic lupus erythematosus
VT	venous thrombosis
VWF	von Willebrand factor
BMI	body mass index
HIV/AIDS	human immunodeficiency virus/acquired immunodeficiency syndrome
mRNA	messenger ribonucleic acid
